# Importância da Artéria Torácica Interna como Fonte de Circulação Colateral em Pacientes com CRM com Síndrome de Leriche

**DOI:** 10.36660/abc.20230780

**Published:** 2024-05-23

**Authors:** Pedro Silvio Farsky, Marcela Dalla Bernardina Sena, Janayna Thaina Rabelato, Ana Cláudia Gomes Pereira Petisco, Renato Tambelini Arnoni

**Affiliations:** 1 Instituto Dante Pazzanese de Cardiologia São Paulo SP Brasil Instituto Dante Pazzanese de Cardiologia, São Paulo, SP – Brasil

**Keywords:** Revascularização, Síndrome de Leriche, Artéria Torácica Interna

## Abstract

Na cirurgia de revascularização do miocárdio (CRM), o uso da artéria torácica interna (ATI), é uma indicação de Classe I para a anastomose com a artéria descendente anterior esquerda (ADA).

A aterosclerose é uma doença sistêmica, além da doença coronariana, um terço dos pacientes possuem doença arterial obstrutiva periférica (DAOP), que é um complicador da CRM. Na Síndrome de Leriche, a ATI pode ser fonte de circulação colateral para artérias abaixo do nível de oclusão. O seu uso inadvertido pode levar a complicações isquêmicas graves nos territórios dependentes.

## Relato de Caso

Mulher, 51 anos, com doença arterial coronariana (DAC) estável com angina Canadian Cardiovascular Society (CCS) classe III canadense e dispneia NYHA III. Hipertensa, dislipidêmica, ex-tabagista, com histórico familiar positivo para DAC e claudicação intermitente para 200 metros. A tentativa de realização de cateterismo pela artéria femoral revelou obstrução da artéria Aorta em seu segmento distal. O acesso radial revelou doença triarterial com função ventricular preservada, sendo a CRM a indicação de tratamento ideal.

**Figura 1:**
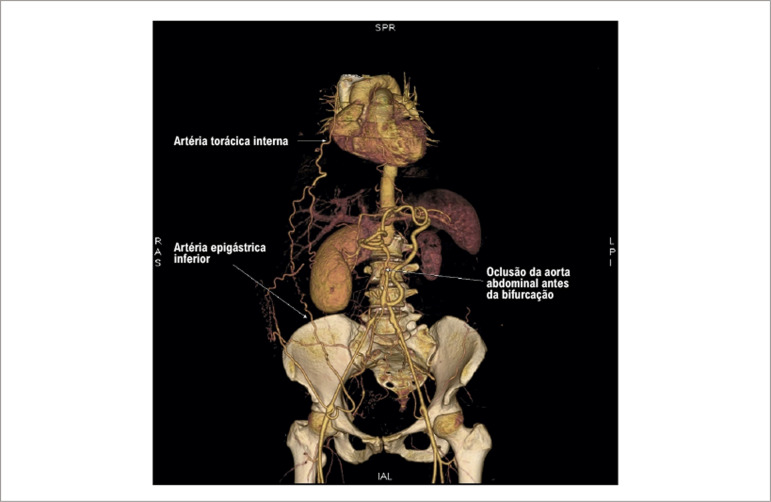
– Renderização de volume 3D. Angiotomografia mostrando a artéria torácica interna como fonte de circulação colateral para a artéria epigástrica inferior.

O pulso femoral não era palpável bilateralmente e o pulso da artéria radial direita era filiforme, além da oclusão da artéria radial esquerda após cateterismo cardíaco. A angiotomografia computadorizada Angio-TC revelou obstrução aórtica total após emergência das artérias renais. Além disso, foi evidenciado que as artérias torácicas internas serviam como fonte de circulação colateral para o enchimento distal da Aorta e das artérias femorais. (Figuras 1 e 2).

**Figura 2:**
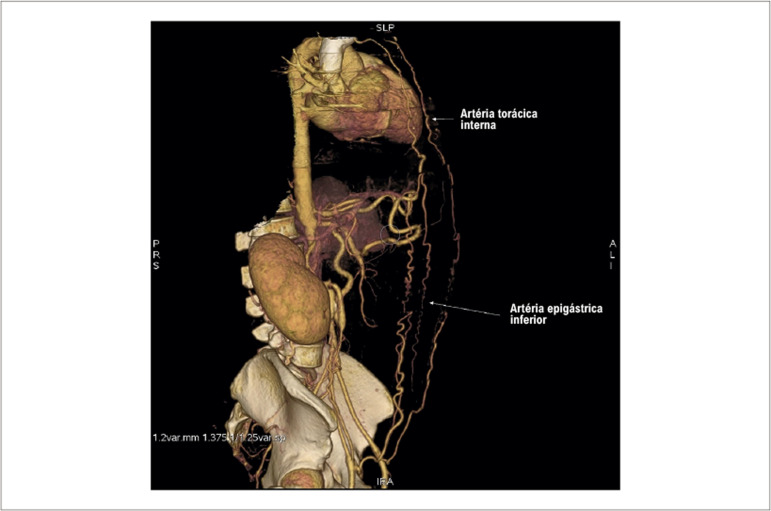
– Renderização de volume 3D. Angiotomografia mostrando a artéria torácica interna como fonte de circulação colateral para a artéria epigástrica inferior.

Neste momento, ficou evidenciada a implausibilidade da utilização das artérias torácicas internas para CRM. Após discussão em Heart Team, foi indicado procedimento percutâneo na coronária direita e artéria descendente anterior (ADA). A artéria circunflexa esquerda ocluída ficou sem intervenção. O procedimento foi bem-sucedido e a paciente recebeu alta no dia seguinte.

## Discussão

Na Síndrome de Leriche, a documentação da origem colateral dos membros inferiores é muito importante nos pacientes com indicação de CRM. Nestes pacientes, a circulação colateral está relacionada ao local da obstrução, quanto maior a oclusão aortoilíaca, menor a chance da origem das colaterais ser proveniente de artérias mesentéricas ou lombares e maior a possibilidade do fluxo ser oriundo das ATIs.^1^

Em pacientes onde a ATI é a principal origem das colaterais das artérias femorais, sua utilização na CRM tem sido associada à isquemia aguda e em alguns casos à isquemia grave de membros inferiores.^2,3^

Dado o maior benefício da ATI à ADA, é imperativo reavaliar a indicação de revascularização do miocárdio, angioplastia ou mesmo manutenção de tratamento clínico otimizado nesses pacientes. Enxertos arteriais alternativos ou enxertos venosos também podem ser usados para substituir as ATIs. Isquemia de membros inferiores em CRM foi descrita anteriormente.^2^ No entanto, foi apenas em 1996 que um estudo da equipe de cirurgia cardiovascular do hospital San Raffaele, em Milão, Itália, descreveu um paciente de 59 anos com Síndrome de Leriche submetido a dupla revascularização da ATI. Observou-se grave isquemia do membro inferior esquerdo, necessitando de fasciotomia e derivação aortobifemoral posterior com enxerto femoral-poplíteo.

Anos mais tarde, Arnold et al., publicaram quatro casos semelhantes, utilizando a ATI esquerda e, posteriormente, realizando a derivação aortobifemoral na mesma internação, com apenas um paciente complicado com amputação de membro.^4^

Ferrer et al.^3^ publicaram três pacientes com diferentes estratégias abordadas, como tratamento clínico otimizado, cirurgia de revascularização apenas com enxertos venosos ou mesmo angioplastia.

Como tratamento alternativo, Bobylev et al.^5^ em 2013 demonstraram a viabilidade de revascularização miocárdica concomitante e derivação aortobifemoral, após circulação extracorpórea.

Não há consenso sobre o tratamento ideal para doença arterial coronariana complexa concomitante e Síndrome de Leriche. Tratamentos alternativos devem ser avaliados. O uso de outros condutos arteriais não está associado à mesma patência em longo prazo que a ATI. O uso de enxerto venoso pode estar associado a um risco aumentado de infecções de feridas nas pernas e a taxas de patência significativamente mais baixas. A angioplastia em pacientes multiarteriais e diabéticos apresenta limitações importantes e deve ser indicada com cautela. A revascularização do miocárdio e a derivação aortobifemoral simultâneas permitem o uso da artéria torácica para proteger o membro inferior, mas são exigentes e podem estar associadas a um risco cirúrgico aumentado. A experiência é limitada nesta área e deve ser uma decisão de um Heart Team.

**Figura 3:**
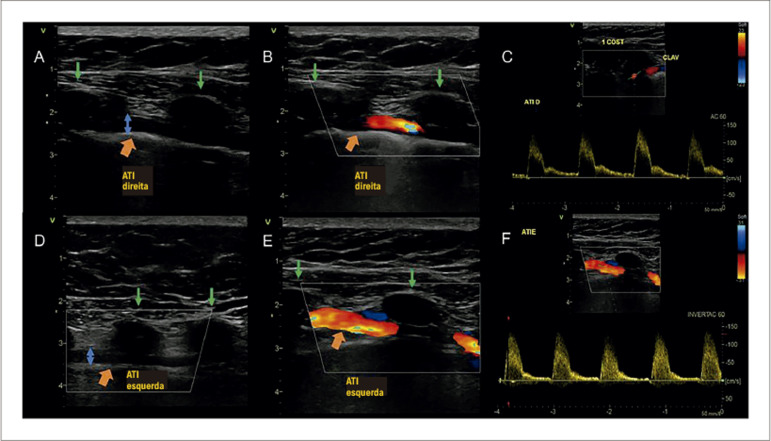
– A e D) ATIs direita e esquerda, visualizadas na ultrassonografia bidimensional; B e E) fluxo nas ATI direita e esquerda ao Doppler colorido; C e F) fluxo nas ATIs ao Doppler espectral. Setas laranja: identificar ATIs na ultrassonografia. Setas azuis: medição do diâmetro da ATI direita (3,6mm) e esquerda (3,3mm). Setas verdes: costelas.

Contudo, é obrigatória a investigação anatômica da ATI como fonte de circulação colateral para o membro inferior. Utilizando angiografia ou angioTC, pode-se avaliar a fonte de circulação colateral para membros inferiores após obstrução aórtica. A ultrassonografia é um método não invasivo capaz de identificar a oclusão aortoilíaca e avaliar a ATI. Diâmetros da ATI maiores que 3,0 mm (ATI direita=3,6mm e ATI esquerda=3,3mm) possivelmente estão relacionados à participação na colateralização para manutenção do fluxo para os membros inferiores (Figura 3).^3^

Essa investigação pode prevenir o uso inadvertido da ATI, que pode culminar em desfechos isquêmicos agudos graves, e proporcionar o melhor tratamento para cada caso.
