# Supplementary material for: Importância da Artéria Torácica Interna como Fonte de Circulação Colateral em Pacientes com CRM com Síndrome de Leriche
Source: Arq Bras Cardiol. 2024 May 23;121(5):e20230780. [Article in Portuguese] doi: 10.36660/abc.20230780 (PMC11164435; doi:10.36660/abc.20230780)
Supplement: Translation

# Translation: Importance of the Internal Thoracic Artery as the Source of Collateral Circulation in CABG Patients with Leriche Syndrome

**DOI:** 10.36660/abc.20230780i

**Authors:** Pedro Silvio Farsky, Marcela Dalla Bernardina Sena, Janayna Thaina Rabelato, Ana Cláudia Gomes Pereira Petisco, Renato Tambelini Arnoni

**Affiliations:** 1 Brazil Instituto Dante Pazzanese de Cardiologia, São Paulo, SP – Brazil

**Keywords:** CABG, Leriche Syndrome, Internal Thoracic Artery

## Abstract

In coronary artery bypass grafting (CABG) the use of the internal thoracic artery (ITA), is a Class I indication for the left anterior descending (LAD) artery.

Atherosclerosis is a systemic disease, and peripheral arterial disease (PAD) is an important complicator of CABG, present in about one-third of the patients with coronary disease. In Leriche Syndrome, ITA can be the source of collateral circulation for arteries below the level of occlusion. Its inadvertent use can lead to serious ischemic complications in the dependent territories.

## Case Report

A 51-year-old female, with stable coronary artery disease (CAD) Canadian Class III and Dyspnea NYHA III. A hypertensive, dyslipidemic, former smoker, with family history, and stable intermittent claudication for 200 meters. A hemodynamic study through the femoral artery revealed aortic obstruction, the exam was interrupted and followed by radial access, that revealed three-vessel disease with preserved ventricular function, so CABG was the preferred choice.

The femoral pulse was bilaterally non-palpable, and the right radial artery pulse was filiform, in addition to new occlusion of the left radial artery after heart cath. Computed Tomography Angiography (CTA) revealed total aortic obstruction after the emergence of the renal arteries. It was also evidenced that bilateral internal thoracic arteries was the source of the collateral circulation for the distal filling (Figures 1 and 2).

**Figure 1:** – 3D volume rendering. Angiotomography showing the internal thoracic artery as a source of collateral circulation to the inferior epigastric artery.

**Figure 2:** – 3D volume rendering. Angiotomography showing the internal thoracic artery as a source of collateral circulation to the inferior epigastric artery.

At this time, the implausibility of using the internal thoracic arteries for CABG was evidenced. After the Heart Team discussion, a percutaneous procedure was indicated to the right coronary and anterior descending artery (LAD). The occluded left circumflex artery was left without intervention. The procedure was successful, and the patient was discharged the next day.

## Discussion

In Leriche Syndrome, the documentation of the collateral branch of the lower limbs is very important in patients with CABG indication. In these syndrome, the collateral circulation is related to the site of obstruction, the higher the aortoiliac occlusion, the lower the chance the origin of collaterals comes from mesenteric or lumbar arteries and the greater the possibility of the flow coming from the ITAs.^1^

In patients where ITA is the main origin of the collateral branch for femoral arteries, their utilization in CABG has been associated with acute ischemia and in some cases with serious ischemia of lower limbs.^2,3^

Given the greater benefit of ITA to LAD, it is imperative to reevaluate the indication of CABG, angioplasty, or even maintenance of optimized clinical treatment in these patients. Alternative arterial grafts or venous grafts can also be used to replace the ITAs. Lower limb ischemia in CABG has been described previously.^2^ However, it was only in 1996 that a letter from the cardiovascular surgery team at the San Raffaele hospital in Milan Italy described a 59-year-old patient with Leriche Syndrome who underwent double ITA revascularization. Serious ischemia of the left lower limb was seen, requiring fasciotomy and posterior aortobifemoral bypass with femoral-popliteal grafts.

Years later, Arnold et al., published four similar cases, using the left ITA and, subsequently, performing the aortobifemoral bypass in the same hospitalization, with only one patient complicated with limb amputation.^4^

Ferrer et al.^3^ published three patients with different strategies addressed, like optimized clinical treatment, revascularization surgery with venous grafts only, or even angioplasty.

As an alternative treatment, Bobylev et al.^5^ in 2013 demonstrated the feasibility of concomitant CABG and aortobifemoral bypass, after extracorporeal circulation.

There is no consensus about the ideal treatment for concomitant complex coronary artery disease and Leriche Syndrome. Alternative treatments should be evaluated. The use of other arterial conduits is not associated with the same long-term patency as the ITA. Use of venous graft may be associated with an increased risk of leg wound infections and significantly lower patency rates. Angioplasty in multivessel disease and diabetic patients has important limitations and should be indicated with caution. Simultaneous CABG and aortobifemoral bypass allow the use of the thoracic artery to protect the lower limb but is demanding and might be associated with increased surgical risk. Experience is limited in this area, and it must be a Heart Team decision.

However, is mandatory the anatomical investigation of the ITA as the source of collateral circulation for the lower limb. Using angiography or CTA, can evaluate the source of collateral circulation to lower limbs after the aortic obstruction. Ultrasonography is a non-invasive method capable of identifying aortoiliac occlusion and evaluating the ITA. ITA diameters greater than 3.0 mm (right ITA=3.6mm and left ITA=3.3mm) are possibly related to participation in collateralization to maintain flow to the lower limbs (figure 3).^3^

**Figure 3:** – A and D) right and left ITAs, seen on bidimensional ultrasound; B and E) flow in the right and left ITA at color Doppler; C and F) flow in the ITAs at spectral Doppler. Orange arrows: identify ITAs on ultrasound. Blue arrows: measurement of the diameter of the right (3.6mm) and left (3.3mm) ITA. Green arrows: ribs.

This investigation can prevent the negligent use of ITA, which can culminate in severe acute ischemic outcomes, and provide the best treatment for each case.
